# Short‐term dual antiplatelet therapy in diabetic patients admitted for acute coronary syndrome treated with a new‐generation drug‐eluting stent

**DOI:** 10.1002/dmrr.3530

**Published:** 2022-04-22

**Authors:** Nousjka P. A. Vranken, Saman Rasoul, Jasper J. P. Luijkx, Tobias F. S. Pustjens, Sonja Postma, Evelien J. Kolkman, Elvin Kedhi, Sodiqur Rifqi, Michael K. Y. Lee, Henning Ebelt, Béla Merkely, Monica Verdoia, Wojtek Wojakowski, Arnoud A. W. J. van ’t Hof, Harry Suryapranata, Giuseppe De Luca

**Affiliations:** ^1^ Department of Cardiology Zuyderland Medical Centre Heerlen The Netherlands; ^2^ Diagram B.V. Zwolle The Netherlands; ^3^ Department of Cardiology Erasmus Hospital Brussels Belgium; ^4^ Department of Cardiology Dr. Kariadi Hospital Semarang Indonesia; ^5^ Department of Cardiology Queen Elizabeth Hospital Hong Kong China; ^6^ Department of Cardiology Catholic Hospital of Johann Nepomuk Erfurt Germany; ^7^ Department of Cardiology Semmelweis University Heart and Vascular Center Budapest Hungary; ^8^ Division of Cardiology Ospedale degli Infermi, ASL Biella Biella Italy; ^9^ Division of Clinical and Experimental Cardiology AOU Sassari, University of Sassari Sassari Italy; ^10^ Department of Cardiology Medical University of Silezia Katowice Poland; ^11^ Department of Cardiology Isala Zwolle The Netherlands; ^12^ Department of Cardiology Radboud University Medical Centre Nijmegen The Netherlands

**Keywords:** acute coronary syndrome, diabetes, dial antiplatelet therapy

## Abstract

**Background:**

The optimal duration of dual antiplatelet therapy (DAPT) in patients with diabetes mellitus (DM) admitted with acute coronary syndrome (ACS) and treated with a drug‐eluting stent (DES) remains unclear. This is a prespecified sub‐study from the Randomised Evaluation of short‐term DUal antiplatelet therapy in patients with acute Coronary syndromE treated with a new generation DES (REDUCE) trial that was designed to determine the efficacy and safety of short‐term versus standard 12 months DAPT in diabetic patients with ACS undergoing percutaneous coronary intervention (PCI) using the COMBO stent.

**Methods:**

In this study we included ACS diabetic patients enroled in the REDUCE trial treated with the COMBO stent and randomly assigned to either 3 or 12 months of DAPT. The primary study endpoint was the composite of all‐cause mortality, myocardial infarction (MI), stent thrombosis (ST), stroke, target vessel revascularisation (TVR), and bleeding complications at 12 and 24 months follow‐up.

**Results:**

A total of 307 diabetic patients were included, of which 162 (52.8%) in the 3 months DAPT group and 145 (47.2%) in the 12 months DAPT group. Patient characteristics, PCI success, and number of stents used were similar in the 3 and 12 months DAPT groups. Occurrence of the primary study endpoint at 12 and 24 months follow‐up was comparable between the two groups (3.1 vs. 3.5%, *p* = 0.865, and 15.8 vs. 14.9%, *p* = 0.824, respectively). Moreover, the prevalence of the specific clinical outcome parameters (all‐cause mortality), MI, ST, stroke, TVR, and bleeding was similar in both study groups.

**Conclusions:**

This sub‐analysis shows similar clinical outcomes following 3 months DAPT as compared to 12 months DAPT in diabetic patients undergoing PCI for ACS using the COMBO stent. These results suggest that, even in this particular subset of patients, short duration of DAPT might be considered safe. Future larger studies are warranted to provide more precise estimations in terms of safety and efficacy of short term DAPT in these high‐risk patients.

## 1 BACKGROUND

In patients presenting with acute coronary syndrome (ACS) due to obstructive coronary artery disease, percutaneous coronary intervention (PCI) is the standard treatment modality. Continuous improvements in stent technology contributed to current patient outcomes, including the relatively low prevalence of stent thrombosis (ST) affecting 0.5%–2% of patients.^1^, ^2^, ^3^ An important factor to prevent complications including ST is the use of dual antiplatelet therapy (DAPT). The beneficial effects of DAPT in terms of lowering the risk of major adverse cardiovascular events (MACE) is well established, and current guidelines therefore recommend the use of DAPT until at least 1 year following the ACS event. On the other hand, bleeding risk and concomitant morbidity and mortality should be taken into account when prescribing or continuing DAPT. Balancing bleeding and thrombotic risk, especially in high risk patients, remains a topic of debate. Although some studies show promising results in patients on short‐term DAPT, contradictory results prevail.^4^, ^5^, ^6^, ^7^

In an attempt to reduce the risk of ST, the COMBO dual therapy stent was introduced. The characteristic property of this new generation stent is the abluminal release of sirolimus (preventing neointima formation and ST) in combination with capturing endothelial progenitor cells (enhancing endothelization), which showed promising results.^8^, ^9^ The COMBO stent was studied to compare 3 months of DAPT versus conventional 12 months of DAPT in the *Randomised Evaluation of short‐term DUal antiplatelet therapy in patients with acute Coronary syndromE treated with a new generation DES* (REDUCE) trial.^10^ The results of the first analysis supported the hypothesis that 3 months is non‐inferior to 12 months DAPT.^11^

Certain comorbidities are known to be associated with an increased risk of thrombotic events, including diabetes. In diabetes mellitus (DM), enhanced platelet reactivity and aggregation as well as hypercoagulability contribute to the pro‐thrombotic state, resulting in an increased risk of ACS as well as the recurrence of thrombotic events.^12^ The independent association with thrombotic events led to inclusion of DM in the so‐called DAPT score, which is designed to aid clinicians in assessing which patient benefits from prolonged DAPT.^13^ This strongly suggests that patients with DM undergoing PCI should be on prolonged rather than short duration of DAPT. On the other hand, bleeding complications are more common among diabetics using DAPT.^14^ To date, no data have been provided on the optimal duration of DAPT in diabetic patients with ACS undergoing DES implantation, hence the aim of the current analysis.

## 2 METHODS

In the REDUCE trial (clinicaltrials.gov, NCT02118870), the COMBO stent (OrbusNeich, Fort Lauderdale, USA) was studied in patients undergoing PCI for ACS.^10^ The REDUCE trial is a multicentre, open‐label, prospective, randomized, and investigator‐initiated trial. The medical ethical committees of all participating study centres approved the study. Detailed data on inclusion and exclusion criteria have already been described.^10^

In the current sub‐analysis, only patients with a previous diagnosis of DM were included. Patients presenting with ACS successfully treated with COMBO stent (postprocedural TIMI 3 flow with residual stenosis <20% based on visual estimation, with no clinical adverse event during hospitalisation) were randomized in a 1:1 fashion to either 3 months or 12 months DAPT. Treatment assignment was performed centrally through a dedicated website as part of the electronic case report form according to computer‐generated random permuted blocks with stratification by site.

Patients were treated with aspirin (ASA) and a P2Y12 inhibitor, with a preference of prasugrel or ticagrelor to clopidogrel. The final choice of the P2Y12 inhibitor was left at the discretion of the treating physician. Patients received DAPT according to their randomization group and continued ASA monotherapy afterwards. In case of an emerging contraindication for ASA, monotherapy with P2Y12 inhibition was allowed.

The primary and secondary study endpoints were the composite of all‐cause mortality, myocardial infarction (MI), ST (the composite of probable and definite ST), stroke, target‐vessel revascularisation (TVR), and bleeding at 12 and 24 months. Myocardial infarction was defined according to the third universal definition,^15^ ST according to the Academic Research Consortium (ARC) definition,^16^ using the combination of the categories ‘‘definite ST’’ and ‘‘probable ST’’ for assessment of event rates. Bleeding was assessed in compliance to the Bleeding Academic Research Consortium (BARC), with BARC categories II, III, and IV included in the current study.^17^ An independent clinical event committee was blinded to the randomization and adjudicated all serious adverse events and determined whether the revascularisation events were related to the index procedure target vessel.

### 2.1 Statistical analysis

Distribution of continuous parameters was assessed for normality. In case of non‐normally distributed parameters, values were expressed as median [25th‐75th percentile] and categorical data as percentages. Mann‐Whitney U or independent samples *t*‐tests were used depending on data distribution. Chi^2^ testing or Fisher's exact test, when appropriate, were used for assessment of categorical variables. The initial sample size calculation was based on a non‐inferiority design, with a power of 80%, a margin for non‐inferiority of 5%.^7^ No sample size calculation was performed for the present substudy.

## 3 RESULTS

Between June 2014 and May 2016, a total of 307 patients with DM, out of 1496 ACS patients included in the REDUCE trial were randomly assigned to either 3 months (*N* = 162, 52.8%) or 12 months (*N* = 145, 47.2%) DAPT. The results showed no significant differences in demographic, clinical characteristics, and outcomes between the 3 and 12 months DAPT groups.

### 3.1 Patient characteristics

Around 26% patients (*n* = 81) were insulin dependent, constituting the 28% and 24% in the 3 and 12 months DAPT groups, respectively. Patient characteristics were similar between the two groups. Females composed of 24% of the study population, and the median age was around 61 years. Presentation with STEMI was observed in more than 40% of patients in both groups (Table 1). Conventional cardiovascular risk factors were present in most patients, with hypertension being the most common with a prevalence of approximately 74%, followed by hypercholesterolaemia, in around 67% of patients. A history of previous revascularisation procedures or stroke was equally prevalent across groups. PCI was performed previously in 14.8% and 18.6% of patients in the 3 and 12 months DAPT groups, respectively (*p* = 0.371). Patients in the 3 months DAPT group showed higher troponin and creatinine kinase values at presentation compared to the 12 months DAPT groups, though not statistically significant.

**TABLE 1:** Baseline patient characteristics

	3 months DAPT (*n* = 162)	12 months DAPT (*n* = 145)	*p*‐value
Age (years)	62.0 (53.0–71.0)	61.0 (52.0–69.0)	0.606
Female gender n(%)	39 (24.1)	35 (24.1)	0.990
BMI (kg/m^2^) median (IQR)	28.5 (24.7–30.8)	27.4 (25.2–30.1)	0.575
Smoker *n*(%)	49 (30.8)	54 (37.8)	0.204
Hypercholesterolaemia *n*(%)	108 (66.7)	98 (67.6)	0.864
Hypertension *n*(%)	120 (74.1)	107 (73.8)	0.955
Prior ACS *n*(%)	29 (17.9)	29 (20.0)	0.639
Prior PCI *n*(%)	24 (14.8)	27 (18.6)	0.371
Prior CABG *n*(%)	8 (4.9)	8 (5.5)	0.820
Prior CVA *n*(%)	4 (2.5)	5 (3.4)	0.612
Presentation with UAP *n*(%)	35 (21.6)	22 (15.3)	0.207
Presentation with NSTEMI *n*(%)	57 (35.2)	63 (43.8)	
Presentation with STEMI *n*(%)	70 (43.2)	59 (41.0)	
hsTnt at presentation (ug/L)	251 (30–1100)	92 (28–469)	0.475
CK at presentation (U/L)	265 (102–814)	209 (104–746)	0.401
LDL (mmol/L)	2.6 [1.9–3.5]	2.6 [2.0–3.5]	0.793

*Note*: Numerical variables are expressed as median (interquartile range). No significant differences were found between the 3 and 12 months groups.

Abbreviations: ACS, acute coronary syndrome; BMI, body mass index; CABG, coronary artery bypass grafting; CK, creatinin kinase; CVA, cerebrovascular accident; DAPT, dual antiplatelet therapy; hsTnt, high sensitivity troponin T; LDL, low density lipoproteins; NSTEMI, non ST‐elevation myocardial infarction; STEMI, ST‐elevation myocardial infarction; UAP, unstable angina pectoris.

### 3.2 Coronary intervention

Single vessel coronary artery disease was the most common finding for the index coronary lesion, resulting in treatment by a single DES to be the most prevalent in this study population (Table 2). Total stent length and diameter did not differ in the 2 study arms. In both groups, all patients showed a complete procedural success, defined as thrombolysis in myocardial infarction grade 3 flow following target vessel PCI.

**TABLE 2:** Coronary angiography results

		3 months DAPT (*n* = 162	12 months DAPT (*n* = 145)	*p*‐value
CAG results	1VD	92 (56.8)	89 (61.4)	0.716
	2VD	49 (30.2)	39 (26.9)	
	3VD	21 (13.0)	17 (11.7)	
Total stent length (mm ± SD)		26.5 ± 14.6	25.4 ± 13	0.49
Stent diameter (mm ± SD)		3 ± 0.39	3.1 ± 0.43	0.06
TIMI flow grade pre‐ PCI	0	33 (20.4)	23 (16.2)	0.97
	1	22 (13.6)	26 (18.3)	
	2	22 (13.6)	23 (16.2)	
	3	85 (52.5)	70 (49.3)	
Number of stents used	1	134 (82.7)	122 (84.1)	0.984
	2	23 (14.2)	19 (13.1)	
	3	3 (1.9)	3 (2.1)	
	4	2 (1.2)	1 (0.7)	
TIMI flow grade post PCI	0	0 (0.0)	0 (0.0)	‐
	1	0 (0.0)	0 (0.0)	
	2	0 (0.0)	0 (0.0)	
	3	162 (100)	145 (100.0)	
DAPT type at discharge (%)				
Clopidogrel		85 (52.5)	80 (55.2)	0.66
Ticagrelor		59 (36.4)	50 (34.5)	
Prasugrel		18 (11.1)	15 (10.3)	

*Note*: Values depicted as *n*(%).

Abbreviations: 1VD, single vessel disease; 2VD, two vessel disease; 3VD, three vessel disease; CAG, coronary angiography; DAPT, dual antiplatelet therapy; TIMI, thrombolysis in myocardial infarction.

### 3.3 Clinical outcomes

At 12 months follow‐up, 3 and 1 patients were lost to follow‐up in the 3 and 12 months DAPT groups, respectively. At maximum follow‐up (24 months), 4 patients were lost to follow‐up in both groups.

**FIGURE 1:**
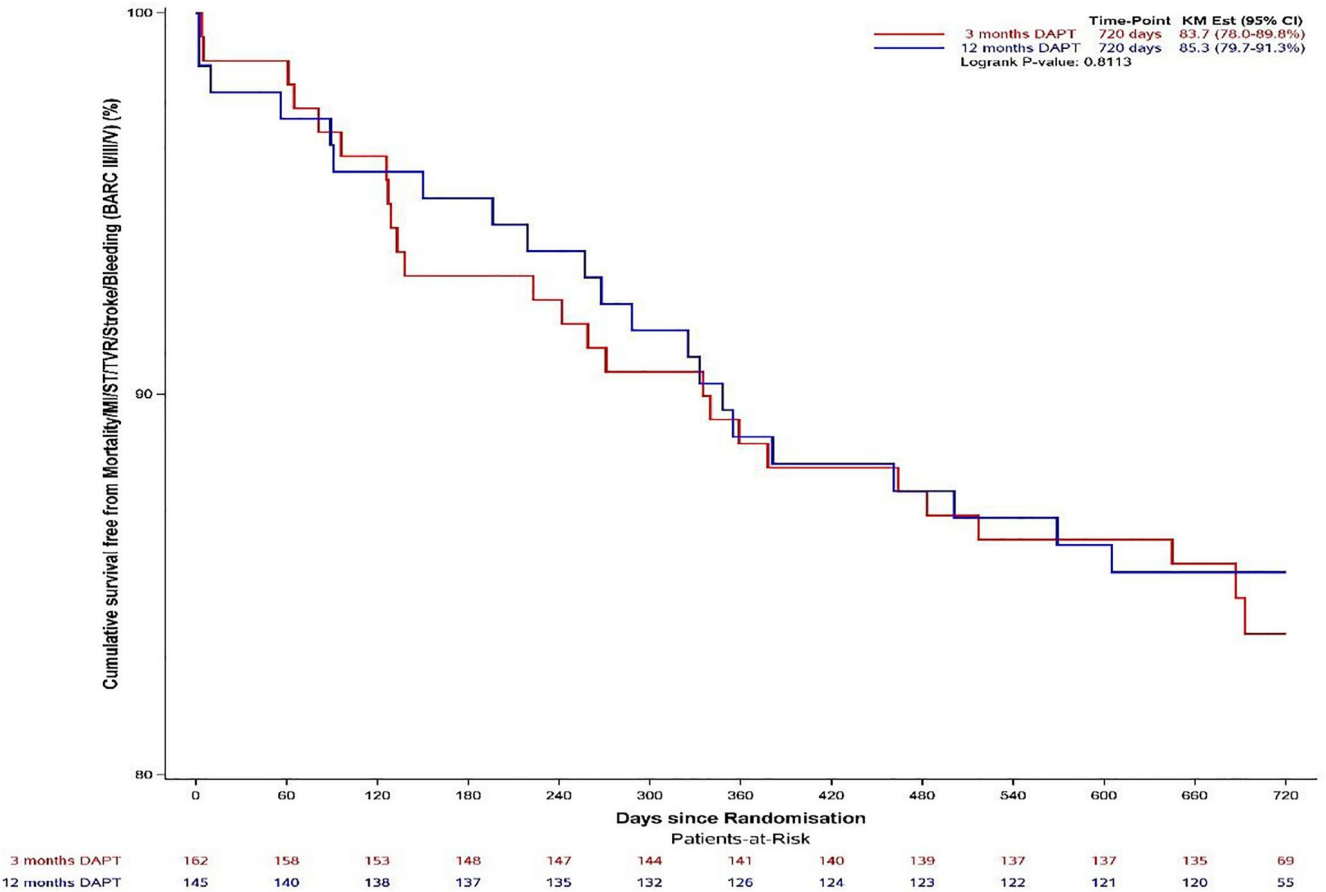
Kaplan–Meier curves illustrating time to composite endpoint in 3 and 12 months DAPT. Red (3 months) and blue (12 months) lines depicting event‐free survival expressed by the Kaplan–Meier estimate. CI, confidence interval; DAPT, dual antiplatelet therapy, KM, Kaplan Meier

In terms of the composite endpoint, no differences were found between the 3 and 12 months DAPT groups (Table 3). More specifically, groups showed no differences in mortality rates, occurrence of bleeding complications, ST, stroke, or recurrent MI (Table 3).At any particular point during follow‐up, the composite endpoint occurred in similar rates in the 3 and 12 months DAPT groups. Comparable results were found for specific outcome parameters, including ST, bleeding, recurrent MI, and mortality (Table 4, Figure 1).

**TABLE 3:** Clinical outcomes

		3 months DAPT	12 months DAPT	*p*‐value
12 months follow‐up	Composite endpoint	18(11.3)	16(11.1)	0.954
	All‐cause mortality	5(3.1)	1(0.7)	0.126
	Cardiac mortality	2(1.3)	0(0.0)	0.177
	ST	1(0.6)	2(1.4)	0.505
	Bleeding	5(3.1)	3(2.1)	0.565
	Stroke	0(0.0)	0(0.0)	‐
	Ischaemic stroke	0(0.0)	0(0.0)	‐
	Recurrent MI	5(3.1)	6(4.2)	0.635
	TVR	6(3.8)	7(4.9)	0.641
24 months follow‐up	Composite endpoint	25(15.8)	21(14.9)	0.824
	All‐cause mortality	9(5.7)	3(2.1)	0.117
	Cardiac mortality	5(3.2)	1(0.7)	0.131
	ST	2(1.3)	2(1.4)	0.909
	Bleeding	7(4.4)	5(3.5)	0.697
	Stroke	0(0.0)	0(0.0)	‐
	Ischaemic stroke	0(0.0)	0(0.0)	‐
	Recurrent MI	8(5.1)	8(5.7)	0.815
	TVR	9(5.7)	8(5.7)	0.993

*Note*: Values depicted as *n*(%).

Abbreviations: DAPT, dual antiplatelet therapy; MI, myocardial infarction; ST, stent thrombosis; TVR, target vessel revascularisation.

**TABLE 4:** Hazard ratios 3 versus 12 months DAPT

	Hazard ratio [95% confidence interval]
Composite endpoint	1.07 [0.60–1.92]
Recurring MI	0.90 [0.34–2.40]
All‐cause mortality	2.75 [0.73–10.14]
ST	0.91 [0.13–0.644]
Bleeding	1.28 [0.41–4.03]

Abbreviations: MI, myocardial infarction; ST, stent thrombosis.

## 4 DISCUSSION

The REDUCE trial was the first study conducted in ACS patients comparing short versus conventional duration of DAPT. The trial showed no differences in clinical outcomes comparing 3 versus 12 months of DAPT.^8^ This prespecified sub‐analysis focussed on diabetic patients admitted with ACS undergoing PCI using the COMBO stent, revealed similar clinical outcome in patients treated with short‐term DAPT compared to those with conventional 12 months DAPT.

Diabetes is a known major risk factor for cardiovascular disease,^18^ and it is estimated that up to 30% of patients undergoing coronary revascularisation are diabetic.^19^ Moreover, DM is associated with less favourable outcomes in ACS patients in terms of coronary revascularisation, illustrated by higher rates of in‐hospital MACE and target lesion revascularisation.^20^, ^21^, ^22^, ^23^ This is confirmed by a recent systematic review by Yuan and Xu, in which the authors concluded that late ST, defined as >30 days following PCI, was more common in diabetic patients.^24^ Definite ST is estimated to occur in 2.1% of patients undergoing PCI in the following 3 years, with a peak incidence in the first 30 days.^25^, ^26^ Until now, only few studies focussed on new generation DES and the duration of DAPT including, and in particular focussing on ACS patients treated with the new adenosine diphosphate receptor antagonists Prasugrel and Ticagrelor. In second generation DES, the incidence of ST has shown to vary and depend on the stent type, amongst other factors. For example, the SPIRIT IV study compared the everolimus‐eluting stent with the paclitaxel‐eluting stent, showing ST incidence rates of 0.3% and 0.8% in 12 months, respectively, while the COMPARE study reported 1.0% and 3.0% with similar stent types and a similar follow‐up duration.^27^, ^28^ Comparable rates of ST were also observed in the complete analysis of the REDUCE trial,^10^ (1.2% vs. 0.4%), which were slightly increased in the present diabetic subpopulation (1.3% and 1.4% in the 3 and 12 months DAPT groups, respectively at 24 months follow‐up). These numbers, in fact, are slightly higher, though still comparable to other studies assessing sirolimus eluting stents, reporting ST in 0%–1.0% of patients in the general population.^24^, ^29^, ^30^

Our results were in line with the previous available literature also in terms of TVR. In a study,^31^ where a polymer‐free biolimus‐coated stent was used in high risk bleeding patients receiving merely 1 month of DAPT, around 5.1% of patients underwent clinically driven TVR at 13 months of follow‐up, which is close to the current 5.7% assessed at 24 months. Conversely, the ONYX ONE trial showed an incidence of TVR of around 17% following 1 month DAPT using either a polymer‐based zotarolimus or polymer‐free biolimus‐coated stent.^32^ Differences in the pharmacologic agent incorporated in the stent or patient characteristics could certainly have played a role in explaining a threefold increased risk of events in this trial. Indeed, the ONYX ONE study included high bleeding risk patients with the majority of cases aged above 74 years, while in the current study subjects had a median age of around 61 with the 3^rd^ quartile at around 70 years old. Nevertheless, the inclusion of a selected ACS population of diabetic patients, in the present analysis, should have been expected to enhance the thrombotic risk,which conversely was not observed.

Apart from an increased risk of reoccurring thrombotic events, diabetic patients are more prone to major bleeding, refraining clinicians from administering more potent antiplatelet therapies in diabetic patients.^14^ Several studies focussed on the efficacy and safety of prolonged DAPT in these high‐risk patients, with contradicting results.^33^, ^34^, ^35^ The ADAPT‐DES study assessed bleeding rates in a large cohort of close to 9000 patients undergoing PCI, who were treated with DAPT using clopidogrel for at least 1 year. The observed bleeding rate at 30 days and 2 years follow‐up was 0.7% and 8.8%, respectively.^36^ Their short‐term bleeding risk was similar to our study, but the long‐term bleeding risk was higher, approximately a two‐fold. The authors did not clarify the BARC criteria for the evaluated bleeding complications, which probably affected these reported rates. Moreover, patients in the ADAPT‐DES study, as in some others, received DAPT for ‘‘at least’’ 12 months, which implies that an unknown proportion of patients who received DAPT for a prolonged period of time, was exposed to an increased risk of bleeding due to prolonged DAPT.

Similar to the current study, the RESET trial assessed outcomes in 3 versus 12 months of DAPT.^37^ No differences were found between groups in terms of ST, minor, or major bleeding complications. The DAPT‐STEMI trial choose a different approach, selecting patients with an uneventful first 6 months after PCI with second generation DES, randomized to either aspirin only (single antiplatelet therapy, SAPT) or DAPT for another 6 months.^38^ In line with current results, the DAPT‐STEMI study reported the SAPT approach to be non‐inferior to DAPT with similar risks of bleeding and ST across study groups. Bleeding, however, consisted merely of BARC type 3 complications. This, in addition to the inclusion of STEMI patients only, which generally represent a younger population, may contribute to explain the relatively low incidence of bleeding complications in the DAPT‐STEMI study (0.5% and 0.9% in the SAPT and DAPT groups, respectively), as compared to the current study (3.0%–4.1% across study groups). Even though we included bleeding complications BARC II to IV, bleeding did not dominate the composite endpoint and the incidence of bleeding was still relatively low. In fact, in the SENIOR trial, a DES and a short duration of DAPT emerged as the most promising strategy among elderly patients undergoing PCI, lowering the rates of ischaemic and bleeding events.^39^

In accordance to these conclusions, the large MASTER‐DAPT trial, including >4000 patients with high‐bleeding risk, reported that 1 month of dual antiplatelet therapy was non‐inferior to the continuation of therapy for at least 2 additional months with regard to the occurrence of net adverse clinical events and major adverse cardiac or cerebral events. However, an abbreviated therapy resulted in a lower incidence of major or clinically relevant non‐major bleeding.^40^

Similar results with a shorter DAPT course have been observed in the large TWILIGHT trial and confirmed in the analysis restricted to ACS patients, where ticagrelor monotherapy after a 3‐month DAPT course, reduced clinically meaningful bleeding events without increasing ischaemic risk as compared with ticagrelor plus aspirin.^41^

Bleeding and ST were also found to be similar across groups in the SMART‐DATE study, that randomized patients to 6 or 12 months DAPT following PCI, using either one of three DES (zotarolimus‐, everolimus‐, or biolimus A9 eluting stent).^42^ However, the 6 months group showed a higher risk of MI compared to the 12 months DAPT group; 24 (1.8%) patients versus 10 (0.8%), hazard ratio 2.41, 95% confidence interval (1.15–5.05), *p* = 0.02. In our study, the MI occurrence was higher, around 5.5% at approximately 24 month follow‐up (Table 3). This might be partially explained by the fact that a previous ACS (prior to study inclusion) was more common in our patient population, between 17.9% and 20% (around 19% in the complete cohort, vs. 2.3% and 1.7% in the study groups included in the SMART‐DATE study). The observed difference in post‐PCI MI occurrence between DAPT groups in our study was found to be non‐significant. This might be due to the study being underpowered to detect differences in individual outcome parameters, as opposed to the composite endpoint.

No outcome difference was also reported in a study restricted to ACS patients, the STOPDAPT2‐ACS trial, concluding that 1 month of dual antiplatelet therapy (DAPT) followed by clopidogrel monotherapy in patients with acute coronary syndrome (ACS) undergoing PCI did not lead to lower rates of cardiovascular and bleeding events compared with 12 months of DAPT.^43^

None of the previous studies reported on the safety of short versus conventional duration of DAPT in diabetic patients, who especially are at risk for reoccurring thrombotic events. The current study showed similar rates of ST and bleeding complications in diabetic patients on short‐term versus conventional duration of DAPT. These results suggest that short‐term DAPT, in combination with the COMBO stent, may be considered safe in this subset of high risk patients.

### 4.1 Considerations and limitations

Only few reports regarding shorter DAPT duration in ACS patients undergoing PCI using DES have been published to date. Some studies, including the current, conclude that shorter duration of DAPT might be safe. A few factors must be considered when interpreting the current and previous study results.

The observed all‐cause mortality rates were low in the current study, with cardiovascular mortality concerning approximately half of the cases. Also stroke and ischaemic stroke occurred in only few patients in the total cohort. The study was not powered to detect differences at such low event rates, these elements of the results should thus be interpreted with caution. The cause of these low mortality rates might be found in the fact that study participants were recruited following successful stenting, thus excluding particular procedural high risk patients, that is, patients with periprocedural complications, patients presenting with out of hospital cardiac arrest due to ACS, and patients who were not considered eligible for PCI. In fact, procedural success, considered as the final TIMI 3 flow, was achieved in the totality of the study population. This might have resulted in underestimated event rates, and to some degree, selection bias. The same accounts for the occurrence of ST, especially at short‐term follow‐up. When assessing complications that occur at relatively low rates, studies usually prefer to express the outcome by means of a composite variable. In this case that consists of (at least) ST, bleeding complications, and mortality, it might result in a lack of power to detect group differences in relevant particular outcome parameters. Future studies, therefore, should focus on recruiting larger cohorts in order to obtain sufficient evidence on differences, or the lack thereof, in clinical outcomes in different modes of DAPT.

Across previous studies published on the duration of DAPT following PCI, there is heterogeneity to be found in study and design characteristics, which makes direct comparison of results challenging. Besides differences in the specific antineoplastic agents incorporated in the different DES, studies use different modes of dual antiplatelet treatment (e.g., clopidogrel, prasugrel, or ticagrelor, by which the preferred P2Y12 inhibitor is mostly left at the discretion of the treating cardiologist), as well as the duration of DAPT in the proposed ‘‘control” groups. Some studies report DAPT of at least 12 months in their conventional DAPT duration group. This might introduce a variety of DAPT durations within the supposed control group, resulting in a variety of (increased) risks of ST and bleeding potentially accompanied by the prolonged use of DAPT.

## 5 CONCLUSION

This prespecified sub‐study showed similar clinical outcomes following short‐term DAPT (3 months) compared to conventional duration of DAPT (12 months) in both diabetic patients with ACS undergoing PCI using the COMBO stent. These results suggest that, even in this particular subset of patients, a short duration of DAPT might be considered safe. Future studies are warranted to provide more precise estimations in terms of safety and efficacy of short‐term DAPT in high risk patients.

## CONFLICT OF INTEREST

The authors declared no conflict of interest.

## ETHICS STATEMENT

The central ethical committee (ISALA Hospital, Zwolle, The Netherlands) initially approved the study. Depending on local regulations, besides the central ethical committee, local ethical committees and competent authorities of each participating centre also approved the study. Our study complies with the Declaration of Helsinki. All patients signed the informed consent before study inclusion.

## CONSENT FOR PUBLICATION

Not applicable.

## AUTHOR CONTRIBUTIONS

Substantial contributions to the conception or design of the work; Data collection; Analysis and interpretation of data for the work; Draughting the work or revising it critically for important intellectual content; Final approval of the version to be published; Agreement to be accountable for all aspects of the work in ensuring that questions related to the accuracy or integrity of any part of the work are appropriately investigated and resolved.

### PEER REVIEW

The peer review history for this article is available at https://publons.com/publon/10.1002/dmrr.3530.

## Data Availability

The data that supports the findings of this study are available in the supplementary material of this article.

## References

[1] De Luca G , Smits P , Hofma SH , et al. Everolimus eluting stent vs first generation drug‐eluting stent in primary angioplasty: a pooled patient‐level meta‐analysis of randomized trials. Drug‐Eluting Stent in Primary Angioplasty (DESERT 3) cooperation. Int J Cardiol. 2017;244:121‐127. 28673736 10.1016/j.ijcard.2017.06.022

[2] De Luca G , Dirksen MT , Spaulding C , et al. Time course, predictors and clinical implications of stent thrombosis following primary angioplasty. Insights from the DESERT cooperation. DESERT cooperation. Thromb Haemostasis. 2013;110(4):826‐833. 23864101 10.1160/TH13-02-0092

[3] De Luca G , Dirksen MT , Spaulding C , et al. Drug‐eluting vs bare‐metal stents in primary angioplasty: a pooled patient‐level meta‐analysis of randomized trials. Drug‐Eluting Stent in Primary Angioplasty (DESERT) Cooperation. Arch Intern Med. 2012;172(8):611‐621. 22529227 10.1001/archinternmed.2012.758

[4] Navarese EP , Andreotti F , Schulze V , et al. Optimal duration of dual antiplatelet therapy after percutaneous coronary intervention with drug eluting stents: meta‐analysis of randomized controlled trials. BMJ. 2015;350:h1618. 25883067 10.1136/bmj.h1618 PMC4410620

[5] Giustino G , Baber U , Sartori S , et al. Duration of dual antiplatelet therapy after drug‐eluting stent implantation: a systematic review and meta‐analysis of randomized controlled trials. J Am Coll Cardiol. 2015;65:1298‐1310. 25681754 10.1016/j.jacc.2015.01.039

[6] Palmerini T , Benedetto U , Bacchi‐Reggiani L , et al. Mortality in patients treated with extended duration dual antiplatelet therapy after drug‐eluting stent implantation: a pairwise and Bayesian network meta‐analysis of randomised trials. Lancet. 2015;385:2371‐2382. 25777667 10.1016/S0140-6736(15)60263-X

[7] Verdoia M , Kedhi E , Suryapranata H , Frati G , Biondi‐Zoccai G , De Luca G . Benefits of short‐term or prolonged as compared to standard 1 year DAPT in patients with acute coronary syndrome treated with drug‐eluting stents: a meta‐analysis of 9 randomized trials. J Thromb Thrombolysis. 2020;50(2):337‐354. 31919736 10.1007/s11239-019-02033-2

[8] Haude M , Lee SW , Worthley SG , et al. The REMEDEE trial: a randomized comparison of a combination sirolimus‐eluting endothelial progenitor cell capture stent with a paclitaxel‐eluting stent. JACC Cardiovasc Interv. 2013;6:334‐343. 23523459 10.1016/j.jcin.2012.10.018

[9] Woudstra P , De Winter RJ , Beijk MA . Next‐generation DES: the COMBO dual therapy stent with Genous endothelial progenitor capturing technology and an abluminal sirolimus matrix. Expet Rev Med Dev. 2014;11:121‐135. 10.1586/17434440.2014.882046 24484431

[10] Camaro C , Damen SAJ , Brouwer MA , et al. Randomized evaluation of short‐term dual antiplatelet therapy in patients with acute coronary syndrome treated with the COMBO dual therapy stent: rationale and design of the REDUCE trial. Am Heart J. 2016;178:37‐44. 27502850 10.1016/j.ahj.2016.04.016

[11] De Luca G , Damen SA , Camaro C , et al. Final results of the randomised evaluation of short‐term dual antiplatelet therapy in patients with acute coronary syndrome treated with a new‐generation stent (REDUCE trial). EuroIntervention. 2019;15(11):e990‐e998. 31422929 10.4244/EIJ-D-19-00539

[12] Vinik AI , Erbas T , Park TS , Nolan R , Pittenger GL . Platelet dysfunction in type 2 diabetes. Diabetes Care. 2001;24:1476‐1485. 11473089 10.2337/diacare.24.8.1476

[13] Yeh RW , Secemsky E , Kereiakes DJ , et al. Development and validation of a prediction rule for benefit and harm of dual antiplatelet therapy beyond one year after percutaneous coronary intervention: an analysis from the randomized Dual Antiplatelet Therapy Study. JAMA. 2016;315(16):1735‐1749. 27022822 10.1001/jama.2016.3775 PMC5408574

[14] Ferreiro JL , Angiolillo DJ . Diabetes and antiplatelet therapy in acute coronary syndrome. Circulation. 2011;123(7):798‐813. 21343595 10.1161/CIRCULATIONAHA.109.913376

[15] Thygesen K , Alpert JS , Jaffe AS , Simoons ML , Chaitman BR , White HD . Joint ESC/ACCF/AHA/WHF task force for the universal definition of myocardial infarction. Third universal definition of myocardial infarction. Circulation. 2012;126:2020‐2035.22923432

[16] Cutlip DE , Windecker S , Mehran R , et al. Clinical end points in coronary stent trials: a case for standardized definitions. Circulation. 2007;115:2344‐2351. 17470709 10.1161/CIRCULATIONAHA.106.685313

[17] Mehran R , Rao SV , Bhatt DL , et al. Standardized bleeding definitions for cardiovascular clinical trials: a consensus report from the Bleeding Academic Research Consortium. Circulation. 2011;123:2736‐2747. 21670242 10.1161/CIRCULATIONAHA.110.009449

[18] Dokken BB . The pathophysiology of cardiovascular disease and diabetes: beyond blood pressure and lipids. Diabetes Spectr. 2008;21(3):160‐165.

[19] Aronson D , Edelman ER . Revascularization for coronary artery disease in diabetes mellitus: angioplasty, stents and coronary artery bypass grafting. Rev Endocr Metab Disord. 2010;11:75‐86. 20221852 10.1007/s11154-010-9135-3 PMC3076727

[20] Sohrabi B , Ghaffari S , Habibzadeh A , Chaichi P . Outcome of diabetic and non‐diabetic patients undergoing successful percutaneous coronary intervention of chronic total occlusion. J Cardiovasc Thorac Res. 2011;3(2):45‐48. 24250951 10.5681/jcvtr.2011.009 PMC3825329

[21] Konigstein M , Ben‐Yehuda O , Smits PC , et al. Outcomes among diabetic patients undergoing percutaneous coronary intervention with contemporary drug‐eluting stents ‐ analysis from the BIONICS randomized trial. JACC Cardiovasc Interv. 2018;11(24):2467‐2476. 30573057 10.1016/j.jcin.2018.09.033

[22] De Luca G , Verdoia M , Savonitto S , et al. Elderly ACS 2 Investigators. Impact of diabetes on clinical outcome among elderly patients with acute coronary syndrome treated with percutaneous coronary intervention: insights from the ELDERLY ACS 2 trial. J Cardiovasc Med. 2020;21(6):453‐459. 10.2459/JCM.0000000000000978 32355067

[23] De Luca G , Dirksen MT , Spaulding C , et al. DESERT cooperation. Impact of diabetes on long‐term outcome after primary angioplasty: insights from the DESERT cooperation. Diabetes Care. 2013;36(4):1020‐1025. 23275351 10.2337/dc12-1507 PMC3609523

[24] Yuan J , Xu GM . Early and late stent thrombosis in patients with versus without diabetes mellitus following percutaneous coronary intervention. Am J Cardiovasc Drugs. 2018;18(6):483‐492. 30132141 10.1007/s40256-018-0295-y

[25] van Werkum JW , Heestermans AA , de Korte FI , et al. Long‐term clinical outcome after a first angiographically confirmed coronary stent thrombosis: an analysis of 431 cases. Circulation. 2009;119(6):828‐834. 19188507 10.1161/CIRCULATIONAHA.108.799403

[26] Buchanan GL , Basavarajaiah S , Chieffo A . Stent thrombosis: incidence, predictors and new technologies. Thrombosis. 2012;2012:956–962. 10.1155/2012/956962 PMC3329679 22577541

[27] Stone GW , Rizvi A , Sudhir K , et al. Randomized comparison of everolimus‐ and paclitaxel‐eluting stents: 2‐year follow‐up from the SPIRIT (clinical evaluation of the XIENCE V everolimus eluting coronary stent system) IV trial. J Am Coll Cardiol. 2011;58(1):19‐25. 21514084 10.1016/j.jacc.2011.02.022

[28] Kedhi E , Joesoef KS , McFadden E , et al. Second‐generation everolimus‐eluting and paclitaxel‐eluting stents in real‐life practice (COMPARE): a randomised trial. Lancet. 2010;375(9710):201‐209. 20060578 10.1016/S0140-6736(09)62127-9

[29] Rasmussen K , Maeng M , Kaltoft A , et al. Efficacy and safety of zotarolimus‐eluting and sirolimus‐eluting coronary stents in routine clinical care (SORT OUT III): a randomised controlled superiority trial. Lancet. 2010;375(9720):1090‐1099. 20231034 10.1016/S0140-6736(10)60208-5

[30] Park DW , Kim YH , Yun SC , et al. Comparison of zotarolimus‐eluting stents with sirolimus‐ and paclitaxel‐eluting stents for coronary revascularization: the ZEST (comparison of the efficacy and safety of zotarolimus‐eluting stent with sirolimus‐eluting and paclitaxel‐eluting stent for coronary lesions) randomized trial. J Am Coll Cardiol. 2010;56(15):1187‐1195. 20883925 10.1016/j.jacc.2010.03.086

[31] Urban P , Meredith IT , Abizaid A , et al. Polymer‐free drug‐coated coronary stents in patients at high bleeding risk. N Engl J Med. 2015;373(21):2038‐2047. 26466021 10.1056/NEJMoa1503943

[32] Windecker S , Latib A , Kedhi E , et al. Polymer‐based or polymer‐free stents in patients at high bleeding risk. N Engl J Med. 2020;382(13):1208‐1218. 32050061 10.1056/NEJMoa1910021

[33] Bundhun PK , Yanamala CM , Huang F . Should a prolonged duration of dual anti‐platelet therapy be recommended to patients with diabetes mellitus following percutaneous coronary intervention? A systematic review and meta‐analysis of 15 studies. BMC Cardiovasc. 2016;16:161. 10.1186/s12872-016-0343-y PMC5006559 27577530

[34] Wang HY , Cai ZX , Yin D , Yang YJ , Song WH , Dou KF . Benefits and risks of prolonged duration dual antiplatelet therapy (clopidogrel and aspirin) after percutaneous coronary intervention in high‐risk patients with diabetes mellitus. Am J Cardiol. 2021;142:14‐24. 33285091 10.1016/j.amjcard.2020.11.043

[35] Rivas Rios JR , Franchi F , Rollini F , Angiolillo DJ . Diabetes and antiplatelet therapy: from bench to bedside. Cardiovasc Diagn Ther. 2018;8(5):594‐609. 30498684 10.21037/cdt.2018.05.09 PMC6232350

[36] Genereux P , Giustino G , Witzenbichler B , et al. Incidence, predictors and impact of post‐discharge bleeding after percutaneous coronary intervention. J Am Coll Cardiol. 2015;66:1036‐1045. 26314532 10.1016/j.jacc.2015.06.1323

[37] Kim BK , Hong MK , Shin DH , et al. A new strategy for discontinuation of dual antiplatelet therapy: the RESET Trial (REal Safety and Efficacy of 3‐month dual antiplatelet Therapy following Endeavor zotarolimus‐eluting stent implantation). J Am Coll Cardiol. 2012;60:1340‐1348. 22999717 10.1016/j.jacc.2012.06.043

[38] Kedhi E , Fabris E , van der Ent M , et al. Six months versus 12 months dual antiplatelet therapy after drug‐eluting stent implantation in ST‐elevation myocardial infarction (DAPT‐STEMI): randomised, multicentre, non‐inferiority trial. BMJ. 2018;363:k3793. 30279197 10.1136/bmj.k3793 PMC6167608

[39] Varenne O , Cook S , Sideris G , et al. Drug‐eluting stents in elderly patients with coronary artery disease (SENIOR): a randomised single‐blind trial. Lancet. 2018;391(10115):41‐50. 29102362 10.1016/S0140-6736(17)32713-7

[40] Valgimigli M , Frigoli E , Heg D , et al, MASTER DAPT Investigators . Dual antiplatelet therapy after PCI in patients at high bleeding risk. N Engl J Med. 2021;385(18):1643‐1655. 34449185 10.1056/NEJMoa2108749

[41] Baber U , Dangas G , Angiolillo DJ , et al. Ticagrelor alone vs. ticagrelor plus aspirin following percutaneous coronary intervention in patients with non‐ST‐segment elevation acute coronary syndromes: TWILIGHT‐ACS. Eur Heart J. 2020;41(37):3533‐3545. 33085967 10.1093/eurheartj/ehaa670

[42] Hahn JY , Song YB , Oh JH , et al. 6‐month versus 12‐month or longer dual antiplatelet therapy after percutaneous coronary intervention in patients with acute coronary syndrome (SMART‐DATE): a randomised, open‐label, non‐inferiority trial. Lancet. 2018;391(10127):1274‐1284. 29544699 10.1016/S0140-6736(18)30493-8

[43] STOPDAPT‐2 ACS: one‐month dual antiplatelet therapy followed by clopidogrel monotherapy in acute coronary syndrome. Presented by Drs. Yuki Obayashi and Ko Yamamoto at the Transcatheter Cardiovascular Therapeutics (TCT) Conference, Orlando, FL, November 5, 2021.

